# Urinary Intestinal Fatty Acid-Binding Protein Can Distinguish Necrotizing Enterocolitis from Sepsis in Early Stage of the Disease

**DOI:** 10.1155/2016/5727312

**Published:** 2016-03-24

**Authors:** Stepan Coufal, Alena Kokesova, Helena Tlaskalova-Hogenova, Jiri Snajdauf, Michal Rygl, Miloslav Kverka

**Affiliations:** ^1^Faculty of Science, Charles University in Prague, 128 43 Prague 2, Czech Republic; ^2^Institute of Microbiology of The Czech Academy of Sciences, v.v.i., 142 20 Prague 4, Czech Republic; ^3^Department of Pediatric Surgery, 2nd Faculty of Medicine, Charles University in Prague and Motol University Hospital, 150 06 Prague 5, Czech Republic; ^4^Institute of Experimental Medicine of The Czech Academy of Sciences, v.v.i., 142 20 Prague 4, Czech Republic

## Abstract

Necrotizing enterocolitis (NEC) is severe disease of gastrointestinal tract, yet its early symptoms are nonspecific, easily interchangeable with sepsis. Therefore, reliable biomarkers for early diagnostics are needed in clinical practice. Here, we analyzed if markers of gut mucosa damage, caspase cleaved cytokeratin 18 (ccCK18) and intestinal fatty acid-binding protein (I-FABP), could be used for differential diagnostics of NEC at early stage of disease. We collected paired serum (at enrollment and week later) and urine (collected for two days in 6 h intervals) samples from 42 patients with suspected NEC. These patients were later divided into NEC (*n* = 24), including 13 after gastrointestinal surgery, and sepsis (*n* = 18) groups using standard criteria. Healthy infants (*n* = 12), without any previous gut surgery, served as controls. Both biomarkers were measured by a commercial ELISA assay. There were no statistically significant differences in serum ccCK18 between NEC and sepsis but NEC patients had significantly higher levels of serum and urinary I-FABP than either sepsis patients or healthy infants. Urinary I-FABP has high sensitivity (81%) and specificity (100%) and can even distinguish NEC from sepsis in patients after surgery. Urinary I-FABP can be used to distinguish NEC from neonatal sepsis, including postoperative one, better than abdominal X-ray.

## 1. Introduction

Necrotizing enterocolitis (NEC) is one of the most common gastrointestinal emergencies in the newborn infant. NEC occurs in 1–3 per 1000 live births, its mortality varies between 15 and 30% and surgical treatment is necessary in 20–40% [1, 2].

NEC accounts for long-term morbidity in survivors of neonatal intensive care and early recognition and proper treatment can improve clinical outcomes [3]. The diagnosis of NEC is based on the combination of clinical, laboratory, and radiologic findings, which are defined by modified Bell's staging criteria [4]. Rapid onset and nonspecific early signs are typical for NEC, so it can be often misdiagnosed as neonatal sepsis. On the other hand, there are only few specific signs, such as* pneumatosis intestinalis* on X-ray or gas in the portal vein on ultrasonography, but these findings appear rather late in the course of the disease so their absence must be interpreted with extreme caution [5]. So there is a strong need for identification of new biomarkers, suitable for early diagnosis of NEC, which would give the opportunity for early intervention.

The best biomarkers should reflect the major steps in early disease pathogenesis. Several defensive mechanisms of innate immune system, such as NK cell response or antimicrobial peptide production, are defective in preterm children, which lead to susceptibility to various microbial infections and mucosal barrier damage [6, 7]. NEC is characterized by destruction of the mucosal layer and by transmural necrosis of the intestinal wall [8]; therefore we start to search for noninvasive test that will reflect this destruction before it is apparent on X-ray. During the enterocyte damage, the cytokeratin 18 (CK18) is released to the circulation. If the cells died preferentially by apoptosis, increased proportion of the caspase cleaved form of CK18 (ccCK18) could be found in the circulation [9]. Interestingly, in animal model of NEC the excessive apoptosis was suggested as a major form of gut epithelium death even before typical necrosis took over [10]. This makes the ccCK18 an interesting target for early NEC diagnosis. Intestinal fatty acid-binding protein (I-FABP) is also released to the circulation during the enterocyte death. This protein constitutes up to 2% of the cytoplasmic protein content in the mature enterocyte [11]. Therefore I-FABP, as a marker of intestinal cell damage, is increased in serum of patients with NEC and sepsis and even in otherwise healthy people after abdominal surgery, trauma, or alcohol consumption [12–15]. Thanks to its small size (14-15 kDa), I-FABP can quickly pass through the kidney to urine [16], which gives an opportunity to measure it noninvasively in urine.

The aim of this study was to investigate the diagnostic value of serum markers of intestinal damage, ccCK18 and I-FABP, in their ability to distinguish NEC from sepsis in early stage of disease.

## 2. Material and Methods

### 2.1. Patients

We enrolled 42 candidate patients with suspected NEC and 12 healthy infants as controls. All of them were recruited from the patients admitted to the Department of Pediatric Surgery of Motol University Hospital, Prague, Czech Republic, between April 2012 and December 2014 (Table 1). The inclusion criteria were stage IA of NEC according to the modified Bell's staging criteria, which are characterized by temperature instability, apnea, lethargy, increased gastric residuals, abdominal distension, and occult blood in stool. These patients were later divided into NEC (*n* = 24), including infants who developed surgery-related NEC (*n* = 13) after the surgery for congenital intestinal malformation (gastroschisis, volvulus, intestinal atresia, anorectal atresia, and Hirschsprung's disease) and sepsis (*n* = 18) using standard criteria for NEC (*pneumatosis intestinalis* on X-ray or presence of gas in portal vein or in peritoneal cavity) or sepsis (suggestive clinical signs, laboratory examination results, and positive blood culture) as published previously [17, 18]. Two patients with sepsis were later excluded from the study, because only one sample was obtained for each of them. All these infants were treated with antibiotics for 7 or more days, according to the recent recommendations [17, 18]. There were no significant differences among groups, except for the birth weight in children suffering from NEC compared with control infants (2.4 ± 0.89 kg versus 3.1 ± 0.90 kg; *p* < 0.05). The study was approved by the Ethics Committee of the Motol University Hospital, and written informed consent was obtained from parents of all infants included in this study.

### 2.2. Sample Collection and Processing

First serum sample was taken at enrollment to the study and the second was taken 7–10 days later, at the last day of antibiotic treatment. Urine samples were collected for two days in 6-hour intervals starting at the time of enrollment to the study. In infants who developed NEC after the surgery for congenital gastrointestinal malformation, the urine was collected in 6 h intervals starting just after the surgery to evaluate the biomarker dynamics in infants before the suspicion of NEC. These samples were analyzed retrospectively in those that developed symptoms. The urine was collected either from urine bag connected to an indwelling catheter or from cotton wool swab placed in the diaper and squeezed through a syringe barrel into a collection tube. The urinary creatinine was measured in each sample immediately after sampling and samples for biomarker analysis were frozen at −20°C.

### 2.3. Enzyme-Linked Immunosorbent Assay (ELISA)

The degree and form of enterocyte death was analyzed by measuring the concentration of total cytokeratin 18 (CK18) and its caspase cleaved form (ccCK18) in serum by M65 or M30 Apoptosense® ELISA (both Peviva, Stockholm, Sweden), respectively. The assay was performed according to the manufacturer's instruction and the concentration of ccCK18 and total CK18 in serum is presented as U/L.

The concentration of I-FABP was measured by Human I-FABP ELISA (Hycult Biotech, Uden, Netherlands), which is certified for both serum and urine. The assay was performed according to the manufacturer's instruction and the I-FABP concentration in serum is presented as ng/mL. To eliminate fluctuation in urine excretion, the urinary I-FABP was normalized to urinary creatinine and it is presented as pg/nmol of creatinine.

### 2.4. Statistical Analysis

The variables were tested for normality by Shapiro-Wilcoxon normality test and the differences between studied groups were analyzed by either Student's *t*-test or Mann-Whitney test. Continuous variables are presented as mean ± standard deviations (SD) and dichotomous variables as percentages. The cutoff levels were calculated as the mean of the group plus 2 SD. Receiving operating characteristic (ROC) analyses were constructed to assess the performance of I-FABP as a predictor of impending NEC and sensitivity, specificity, likelihood ratio, positive predictive value, and negative predictive value were calculated to show diagnostic utility of this approach. Statistical analyses were performed using GraphPad Prism version 6.0 (GraphPad Software, GraphPad Software, San Diego, CA, USA).

## 3. Results

### 3.1. Serum Cytokeratin 18

**Figure 1:**
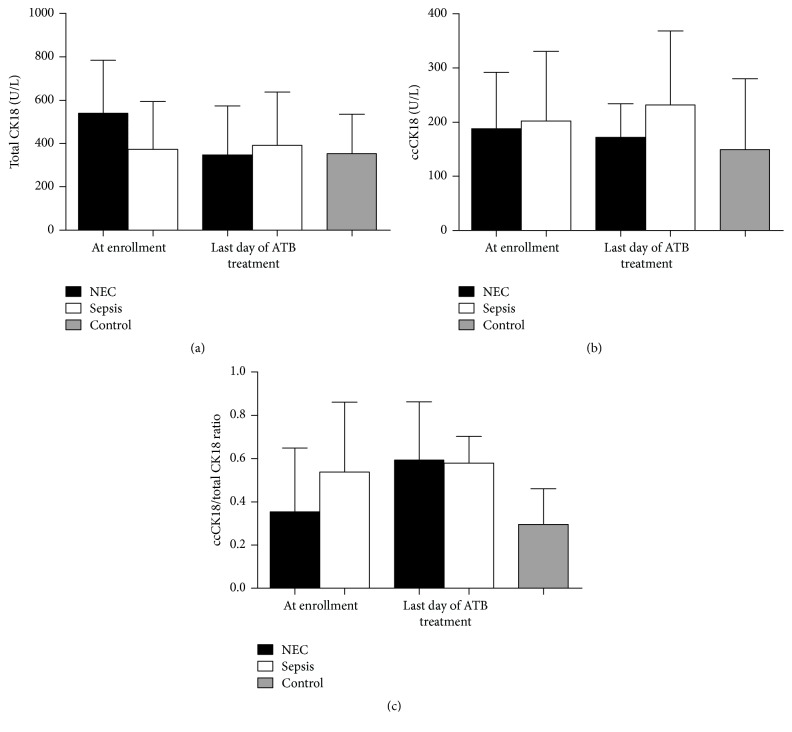
The analysis of cytokeratin 18 (CK18) in serum. (a) The differences between NEC and sepsis in caspase cleaved CK18 (ccCK18) (*p* = 0.7606, Mann-Whitney test), (b) total CK18 (*p* = 0.2317, Mann-Whitney test), and (c) ccCK18/total CK18 ratio (*p* = 0.1783, Mann-Whitney test), at the time of enrollment and last day of antibiotic (ATB) treatment.

There were no statistically significant differences in ccCK18 or total CK18 between NEC and sepsis (Figures 1(a) and 1(b)). At the time of enrollment, there was slightly elevated total CK18 and slightly decreased ccCK18/CK18 ratio in NEC as compared to sepsis suggesting higher proportion of necrotic cell death in NEC patients (Figure 1(c)). These differences were, however, not statistically significant.

### 3.2. Serum I-FABP

**Figure 2:**
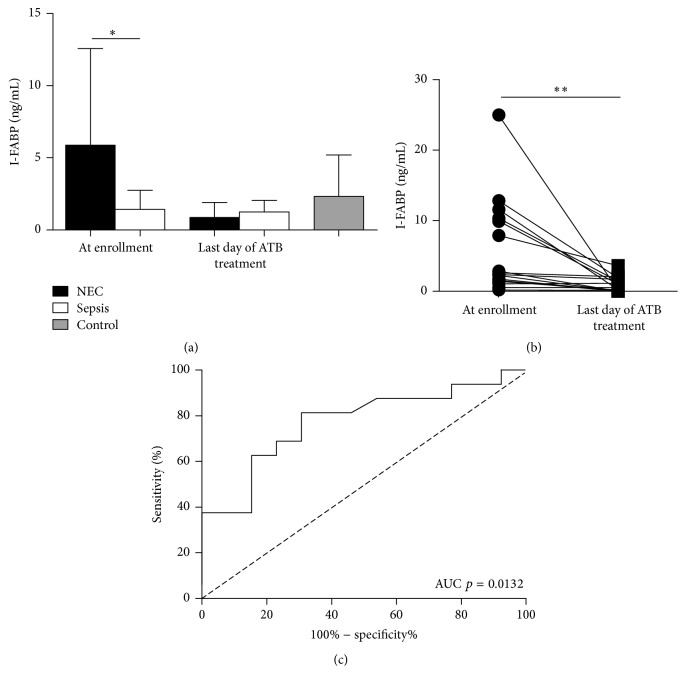
The analysis of I-FABP in serum. (a) The comparison of I-FABP for patients with NEC and sepsis or healthy patients at the time of enrollment (^*∗*^ *p* < 0.05, unpaired Student's *t*-test with Welch's correction), (b) the comparison of I-FABP in patients with NEC at the time of enrollment and last day of ATB treatment (^*∗∗*^ *p* < 0.01, Paired Student's *t*-test), and (c) the ROC curve analysis using I-FABP levels in NEC and sepsis group at the time of admission.

At the time of enrollment, patients who developed NEC had significantly higher concentrations of I-FABP than patients who developed sepsis (Figure 2(a)). At the end of antibiotic therapy, the levels of I-FABP in NEC patients decreased significantly, reaching low levels typical for patients with sepsis or healthy controls (Figure 2(b)). The ROC curve analysis revealed that serum I-FABP is suitable biomarker for distinguishing NEC from sepsis (LR+ = 4.75 LR− = 0.67 and optimal cutoff = 4.1 ng/mL) (Figure 2(c)). There were no significant differences between spontaneous and surgery-related NEC (*p* = 0.60, data not shown).

### 3.3. Analysis of I-FABP in Urine

**Figure 3:**
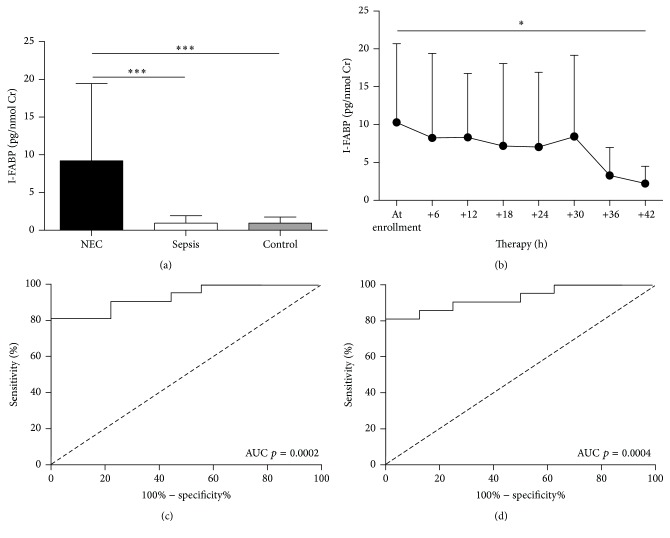
The analysis of I-FABP levels in urine. (a) The I-FABP levels in first twelve hours from the time of enrollment (^*∗∗∗*^ *p* < 0.001, Mann-Whitney test) and (b) the dynamics of I-FABP in urine in NEC patients (^*∗*^ *p* < 0.05, paired Student's *t*-test). The ROC curve analysis using I-FABP levels in (c) NEC and sepsis or (d) NEC and controls.

During the first twelve hours after enrollment, patients who developed NEC had significantly higher concentrations of I-FABP then either those who developed sepsis or healthy infants (Figure 3(a)). There were no significant differences between spontaneous and surgery-related NEC (*p* = 0.80, data not shown). The levels of urinary I-FABP in patients with sepsis were indistinguishable from these found in control infants. The continuous sampling of urine showed that urinary I-FABP levels decrease during the therapy (Figure 3(b)). Urinary I-FABP is, therefore, capable of distinguishing NEC from either sepsis (Figure 3(c)) or healthy infants (Figure 3(d)). All control infants had the level of I-FABP under the calculated cutoff level 2.52 pg/nmol creatinine. Although the levels of urinary I-FABP were significantly higher in patients with stage III NEC than in patients with stage II NEC (data not shown) in the first twelve hours after enrollment, it cannot distinguish between surgical (stage IIIB) and medical (stages IIIA and below) NEC (AUC *p* = 0.18).

### 3.4. Diagnostic Performance of I-FABP ELISA

At the time of enrollment, analysis of urinary I-FABP had higher sensitivity and higher negative predictive value for NEC than either* pneumatosis intestinalis* on X-ray or gas in portal vein on ultrasound (Table 2). When urinary I-FABP analysis was combined with imagine methods the sensitivity and negative predictive values for NEC at the time of first symptoms increased to 91% and 89%, respectively. This approach revealed 9 (33%) radiologically and ultrasonographically negative patients who later developed NEC.

### 3.5. Urinary I-FABP in Diagnosis of Surgery-Related NEC

**Figure 4:**
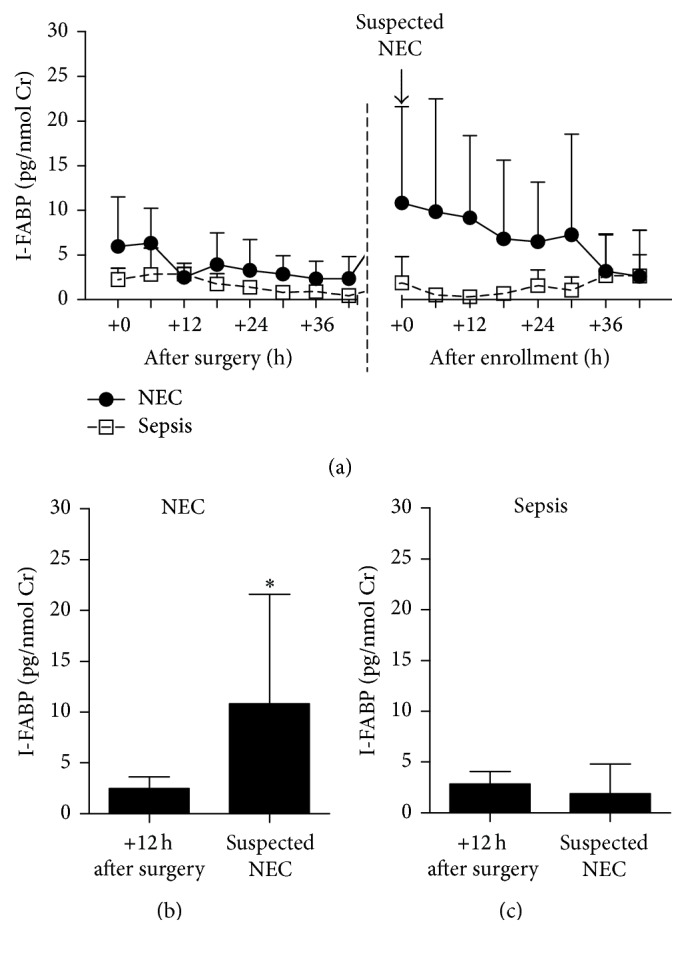
The urinary I-FABP in patients that developed NEC or sepsis after surgery for congenital intestinal malformation. (a) The dynamics of urinary I-FABP. Change in urinary I-FABP between time 12 h after surgery and time of suspected NEC (^*∗*^ *p* < 0.05, Wilcoxon matched-pairs signed-rank test) in (b) NEC and (c) sepsis patients.

Patients who developed NEC after the surgery for congenital intestinal malformation showed rapid increase in urinary I-FABP from the time point of 12 h after surgery to the time of suspected NEC (Figure 4). This increase was not present in infants who later developed sepsis.

## 4. Discussion

Necrotizing enterocolitis is one of the most severe diseases of infant's gastrointestinal tract (GIT). It is characterized by unexpected onset and very rapid progression with the risk of gut perforation and the infant's death. The clinical signs are in the early stage nonspecific and easily interchangeable with other GIT disorders or sepsis.

The diagnosis of NEC is based on presence of clinical symptoms (i.e., abdominal distension and blood in stool), radiologic or sonographic findings of* pneumatosis intestinalis* or gas in portal vein, and in most severe cases presence of gas in peritoneal cavity. Unfortunately, both imagine methods have very low sensitivity and negative predictive value and uncover mainly advanced cases of NEC [19]. Therefore, many NEC cases are missed at the initial submission to the hospital and the newborns with suspected NEC being subjected to harmful effects of ionizing radiation by numerous abdominal X-rays [20]. Early diagnosis of NEC allows more efficient intervention, consisting of cessation of enteral feeding, administration of broad spectrum antibiotics, and supportive care, which has major impact on the disease prognosis [21–23].

Since the gut barrier disruption has crucial role in the early steps of NEC pathogenesis, we analyzed markers of gut barrier disruption, ccCK18 or I-FABP in serum and urine, as possible early biomarkers for NEC and its distinction from sepsis. Although coagulative necrosis of the gut wall is the major hallmark of NEC, it may be preceded with excessive apoptosis of gut epithelial cells, as reported before in animal model of NEC [10]. Therefore, we measured ccCK18 and CK18 as a marker of necrosis and apoptosis of gut epithelium. We did not find any significant differences in ccCK18 and total CK18 concentrations between NEC and sepsis groups. We found only slightly increased proportion of epithelium necrosis at enrollment in infants with NEC, which reflects the major histopathologic feature of the disease [8, 24, 25], but this difference was not statistically significant.

Increased serum concentration of I-FABP as possible biomarker of gut epithelium damage during NEC was first noted in animal model [26]. Later studies in humans found that serum I-FABP can distinguish infants with NEC from healthy preterm infants and that I-FABP levels correlate with NEC severity [27, 28]. Recent meta-analysis concluded that serum I-FABP is a valid biomarker for NEC that can significantly decrease the high false negative rates of current diagnostic procedures [29]. But since increase in I-FABP was described also in sepsis [30], we decided to use serum I-FABP to distinguish NEC from sepsis. We found that I-FABP levels were significantly higher in patients with NEC than those with sepsis at the time of recruitment to the study but normalized after the successful treatment. Moreover, we found that I-FABP levels can distinguish NEC stage II and NEC stage III in the first 12 h after the suspicion of NEC, although it cannot make more clinically relevant distinction between medical and surgical NEC. Nevertheless, recent study demonstrated that length of bowel resection in surgical NEC correlates with I-FABP levels at disease onset, suggesting that the I-FABP levels mirror the degree of intestinal damage [31].

Thanks to its small molecule, I-FABP can be easily measured in urine, thus sparing the infant from unnecessary blood draw. The noninvasive means of its collection is advantageous, because the invasive diagnostic procedures may contribute to adverse neurocognitive outcome [32]. The previous studies showed that urinary I-FABP could be used to distinguish patients with NEC from healthy newborns [16, 33, 34]. In this study we found that I-FABP was significantly higher in infants who developed NEC than in either those who developed sepsis or control infants. Furthermore, the levels of I-FABP in infants with sepsis and control infants were similar. Currently, the gold standard for NEC diagnosis is abdominal X-ray, which has very low sensitivity and negative predictive value [19]. Urinary I-FABP reached 100% positive predictive value, which is similar as that reached by diagnostic gold standard. More importantly, it revealed 9 (33%) radiologically and ultrasonographically negative patients that later developed NEC, thus reaching sensitivity of 81% as compared to either healthy controls or sepsis patients. When standard imagine methods during the admission to the hospital were supplemented with urinary I-FABP analysis, it significantly raised the sensitivity (91%) and negative predictive value (89%) of such combined diagnostic approach, while keeping the high specificity and positive predictive value (both 100%).

Both NEC and sepsis are common complications of surgery for congenital intestinal malformation. The development of NEC is in these cases probably driven by decreased mesenteric blood flow to the intestine. On the other hand, any breach of abdominal wall in infants has some risk of sepsis; its incidence is 8% for laparotomy without enterotomy or 20% for laparotomy with enterotomy [35]. And since even uncomplicated surgery may lead to increase in urinary I-FABP [12, 36, 37], we measured its levels in patients with surgery-related NEC and sepsis. We found that the I-FABP concentration in urine significantly increased in the time of suspected NEC but only in patients who developed NEC later, but not in those who developed sepsis. Therefore, the diagnostic power of urinary I-FABP was not hindered by previous surgery.

## 5. Conclusions

These results show that urinary I-FABP can be used to distinguish NEC from neonatal sepsis, including postoperative one, better than current gold standard (abdominal X-ray). This is very important for the clinician at the moment when the clinical symptoms of NEC are nonspecific and when NEC can be confused with neonatal sepsis. The addition of this harmless and noninvasive examination to the standard X-ray significantly increases the sensitivity and negative predictive value of such approach in the NEC diagnosis.

## Figures and Tables

**Table 1:** Patients' demographics. The data are expressed as number of cases (%) or mean ± standard deviation.

	NEC	Sepsis	Control
Number of infants	24	16	12
NEC stage II	11 (45.8%)	—	—
NEC stage III	13 (54.2%)	—	—
Sex, female	7 (29.2%)	6 (37.5%)	6 (50.0%)
Gestational age (weeks)	35.5 ± 3.3	35.9 ± 4.2	37.8 ± 2.7
Birth weight (kg)	2.4 ± 0.9	2.7 ± 0.9	3.1 ± 0.9
Delivery by cesarean section	14 (58.3%)	5 (31.3%)	6 (50.0%)
Birth asphyxia	10 (41.7%)	5 (31.3%)	2 (16.7%)
Congenital heart disease	4 (16.7%)	0 (0.0%)	0 (0.0%)
Postnatal age at disease evaluation (day)	18.3 ± 30.5	19.9 ± 31.8	13.6 ± 11.4

**Table 2:** Diagnostic performance of standard X-ray (gold standard), ultrasound, and serum or urinary I-FABP as analyzed in this study. Imagine methods refer collectively to the X-ray and ultrasound (USG).

NEC × disease	Imagine methods		Serum I-FABP		Urinary I-FABP		Imagine methods + urinary I-FABP
	X-ray	USG	NEC versus sepsis	NEC versus control	NEC versus sepsis	NEC versus control	
Sensitivity	41%	29%	38%	31%	81%	81%	91%
Specificity	100%	100%	92%	100%	100%	100%	100%
Positive predictive value	100%	100%	86%	100%	100%	100%	100%
Negative predictive value	53%	48%	55%	31%	69%	67%	89%

## Abbreviations

NEC: Necrotizing enterocolitis
I-FABP: Intestinal fatty acid-binding protein
CK18: Cytokeratin 18
ccCK18: Caspase cleaved cytokeratin 18
USG: Ultrasonography
SD: Standard deviation
ROC: Receiving operating characteristic
PPV: Positive predictive value
NPV: Negative predictive value
AUC: Area under the curve
ELISA: Enzyme-linked immunosorbent assay
GIT: Gastrointestinal tract
LI: Likelihood ratio.

## References

[1] Yee W. H., Soraisham A. S., Shah V. S. (2012). Incidence and timing of presentation of necrotizing enterocolitis in preterm infants. *Pediatrics*.

[2] Holman R. C., Stoll B. J., Curns A. T., Yorita K. L., Steiner C. A., Schonberger L. B. (2006). Necrotising enterocolitis hospitalisations among neonates in the United States. *Paediatric and Perinatal Epidemiology*.

[3] Eltayeb A. A., Mostafa M. M., Ibrahim N. H., Eltayeb A. A. (2010). The role of surgery in management of necrotizing enterocolitis. *International Journal of Surgery*.

[4] Walsh M. C., Kliegman R. M. (1986). Necrotizing enterocolitis: treatment based on staging criteria. *Pediatric Clinics of North America*.

[5] Shanbhogue L. K. R., Tam P. K. H., Lloyd D. A. (1991). Necrotizing enterocolitis following operation in the neonatal period. *British Journal of Surgery*.

[6] Li J., Li H., Mao H. (2013). Impaired NK cell antiviral cytokine response against influenza virus in small-for-gestational-age neonates. *Cellular and Molecular Immunology*.

[7] Richter M., Topf H.-G., Gröschl M. (2010). Influence of gestational age, cesarean section, and type of feeding on fecal human *β*-defensin 2 and tumor necrosis factor-*α*. *Journal of Pediatric Gastroenterology and Nutrition*.

[8] Ballance W. A., Dahms B. B., Shenker N., Kliegman R. M. (1990). Pathology of neonatal necrotizing enterocolitis: a ten-year experience. *The Journal of Pediatrics*.

[9] Leers M. P. G., Kölgen W., Björklund V. (1999). Immunocytochemical detection and mapping of a cytokeratin 18 neo-epitope exposed during early apoptosis. *Journal of Pathology*.

[10] Jilling T., Lu J., Jackson M., Caplan M. S. (2004). Intestinal epithelial apoptosis initiates gross bowel necrosis in an experimental rat model of neonatal necrotizing enterocolitis. *Pediatric Research*.

[11] Pelsers M. M. A. L., Namiot Z., Kisielewski W. (2003). Intestinal-type and liver-type fatty acid-binding protein in the intestine. Tissue distribution and clinical utility. *Clinical Biochemistry*.

[12] Bingold T. M., Franck K., Holzer K. (2015). Intestinal fatty acid binding protein: a sensitive marker in abdominal surgery and abdominal infection. *Surgical Infections*.

[13] de Jong W. J., Cleveringa A. M., Greijdanus B., Meyer P., Heineman E., Hulscher J. B. (2015). The effect of acute alcohol intoxication on gut wall integrity in healthy male volunteers; a randomized controlled trial. *Alcohol*.

[14] Machado M. C. E. C., Barbeiro H. V. I., Pinheiro da Silva F., de Souza H. P. O. (2012). Circulating fatty acid binding protein as a marker of intestinal failure in septic patients. *Critical Care*.

[15] Thuijls G., Van Wijck K., Grootjans J. (2011). Early diagnosis of intestinal ischemia using urinary and plasma fatty acid binding proteins. *Annals of Surgery*.

[16] Derikx J. P. M., Evennett N. J., Degraeuwe P. L. J. (2007). Urine based detection of intestinal mucosal cell damage in neonates with suspected necrotising enterocolitis. *Gut*.

[17] Young Infants Clinical Signs Study Group (2008). Clinical signs that predict severe illness in children under age 2 months: a multicentre study. *The Lancet*.

[18] Strunk T., Doherty D., Jacques A. (2012). Histologic chorioamnionitis is associated with reduced risk of late-onset sepsis in preterm infants. *Pediatrics*.

[19] Tam A. L., Camberos A., Applebaum H. (2002). Surgical decision making in necrotizing enterocolitis and focal intestinal perforation: Predictive value of radiologic findings. *Journal of Pediatric Surgery*.

[20] Mazrani W., McHugh K., Marsden P. J. (2007). The radiation burden of radiological investigations. *Archives of Disease in Childhood*.

[21] Bell M. J., Ternberg J. L., Feigin R. D. (1978). Neonatal necrotizing enterocolitis. Therapeutic decisions based upon clinical staging. *Annals of Surgery*.

[22] Lin P. W., Stoll B. J. (2006). Necrotising enterocolitis. *The Lancet*.

[23] Ricketts R. R. (1984). Surgical therapy for necrotizing enterocolitis. *Annals of Surgery*.

[24] Kliegman R. M., Fanaroff A. A. (1984). Necrotizing enterocolitis. *The New England Journal of Medicine*.

[25] McElroy S. J., Underwood M. A., Sherman M. P. (2012). Paneth cells and necrotizing enterocolitis: a novel hypothesis for disease pathogenesis. *Neonatology*.

[26] Gollin G., Marks W. H. (1993). Elevation of circulating intestinal fatty acid binding protein in a luminal contents-initiated model of NEC. *Journal of Pediatric Surgery*.

[27] Aydemir C., Dilli D., Oguz S. S. (2011). Serum intestinal fatty acid binding protein level for early diagnosis and prediction of severity of necrotizing enterocolitis. *Early Human Development*.

[28] Edelson M. B., Sonnino R. E., Bagwell C. E., Lieberman J. M., Marks W. H., Rozycki H. J. (1999). Plasma intestinal fatty acid binding protein in neonates with necrotizing entercolitis: a pilot study. *Journal of Pediatric Surgery*.

[29] Cheng S., Yu J., Zhou M., Tu Y., Lu Q. (2015). Serologic intestinal-fatty acid binding protein in necrotizing enterocolitis diagnosis: a meta-analysis. *BioMed Research International*.

[30] Lieberman J. M., Sacchettini J., Marks C., Marks W. H. (1997). Human intestinal fatty acid binding protein: report of an assay with studies in normal volunteers and intestinal ischemia. *Surgery*.

[31] Heida F. H., Hulscher J. B. F., Schurink M. (2015). Intestinal fatty acid-binding protein levels in Necrotizing Enterocolitis correlate with extent of necrotic bowel: results from a multicenter study. *Journal of Pediatric Surgery*.

[32] Vinall J., Miller S. P., Bjornson B. H. (2014). Invasive procedures in preterm children: brain and cognitive development at school age. *Pediatrics*.

[33] Evennett N. J., Hall N. J., Pierro A., Eaton S. (2010). Urinary intestinal fatty acid-binding protein concentration predicts extent of disease in necrotizing enterocolitis. *Journal of Pediatric Surgery*.

[34] Mannoia K., Boskovic D. S., Slater L., Plank M. S., Angeles D. M., Gollin G. (2011). Necrotizing enterocolitis is associated with neonatal intestinal injury. *Journal of Pediatric Surgery*.

[35] Kessler U., Ebneter M., Zachariou Z., Berger S. (2009). Postoperative sepsis in infants below 6 months of age. *World Journal of Pediatrics*.

[36] Derikx J. P. M., van Waardenburg D. A., Thuijls G. (2008). New insight in loss of gut barrier during major non-abdominal surgery. *PLoS ONE*.

[37] Kittaka H., Akimoto H., Takeshita H. (2014). Usefulness of intestinal fatty acid-binding protein in predicting strangulated small bowel obstruction. *PLoS ONE*.

